# A novel cross-validated machine learning based Alertix-Cancer Risk Index for early detection of canine malignancies

**DOI:** 10.3389/fvets.2025.1570106

**Published:** 2025-04-25

**Authors:** Hanan Sharif, Reza Arabi Belaghi, Kiran Kumar Jagarlamudi, Sara Saellström, Liya Wang, Henrik Rönnberg, Staffan Eriksson

**Affiliations:** ^1^Alertix Veterinary Diagnostics, Stockholm, Sweden; ^2^Department of Animal Biosciences, Swedish University of Agricultural Sciences, Uppsala, Sweden; ^3^Department of Energy and Technology, Swedish Agricultural University, Uppsala, Sweden; ^4^Department of Clinical Sciences, Swedish University of Agricultural Sciences, Uppsala, Sweden

**Keywords:** canine TK1 ELISA, solid tumors, monoclonal antibody, serum TK1 concentration, cCRP, canine lymphoma, gradient boosting algorithm, machine learning models

## Abstract

**Introduction:**

The demand for non-invasive tumor biomarkers in veterinary field has recently grown significantly. Thymidine kinase 1 (TK1) is one of the non-invasive proliferation biomarkers that has been used for diagnosis and treatment monitoring of different canine malignancies. However, recent studies showed that the combination of TK1 with inflammatory biomarkers such as canine C-reactive protein (cCRP) can enhance the sensitivity for early tumor detection. Herein, we developed a machine learning (ML) model, i.e., Alertix-Cancer Risk Index (Alertix-CRI) which incorporates canine TK1 protein, CRP levels in conjunction with an age factor.

**Methods:**

A total of 287 serum samples were included in this study, consisting of 67 healthy dogs and dogs with different tumors (i.e., T-cell lymphoma *n* = 24, B-cell lymphoma *n* = 29, histiocytic sarcoma *n* = 47, hemangiosarcoma *n* = 26, osteosarcoma *n* = 26, mastocytoma *n* = 40, and mammary tumors *n* = 28). Serum TK1 protein levels were measured using TK1-ELISA and cCRP levels by a quantitative ELISA. The whole data set was divided as training (70%) and validation (30%). The Alertix-Cancer Risk Index (Alertix-CRI) is a generalized boosted regression model (GBM) with high accuracy in the training set and further validation was carried out with the same model.

**Results:**

Both the TK1-ELISA and cCRP levels were significantly higher in the tumor group compared to healthy controls (*p* < 0.0001). For overall tumors, the ROC curve analysis showed that TK1-ELISA has similar sensitivity as cCRP (54% vs. 51%) at a specificity of 95%. However, the Alertix-CRI for all malignancies showed an area under the curve (AUC) of 0.98, demonstrating very high discriminatory capacity, with a sensitivity of 90% and a specificity of 97%.

**Conclusion:**

These results demonstrate that the novel Alertix-CRI could be used as a decision-support tool helping clinicians to early differentiate dogs with malignant diseases from healthy. Additionally, these findings would facilitate the advancement of more precise and dependable diagnostic tools for early cancer detection and therapy monitoring within the realm of veterinary medicine.

## Introduction

In veterinary oncology, the detection and treatment assessment of cancer pose unique challenges. To evaluate dogs suspected of having cancer, veterinarians typically employ multiple invasive diagnostic approaches, e.g., biopsies and in certain cases, exploratory surgery and/or endoscopy, which often take long to analyse and interpret. Presently, the focus lies in identifying suitable diagnostic, prognostic, and predictive biomarkers for different malignancies in veterinary medicine using non-invasive and inexpensive sampling techniques. Ideally the tumor biomarker should have high accuracy that would allow determination of the disease at an early stage, predict response to treatment and monitoring progression in an inexpensive way (1).

Thymidine kinase 1 (TK1) is a pyrimidine salvage pathway enzyme and it levels fluctuates during the different phases of the cell cycle. Elevated levels of TK1 in the blood has been associated with various types of cancers, including lymphoma, leukemia, and solid tumors and higher levels of TK1 correlates with advanced stage of disease as well as poor prognosis (2). Several earlier studies have focused on dogs with hematological malignancies, demonstrating the usefulness of measuring serum TK1 activity in monitoring disease progression within the veterinary field (3–6). However, limited research has been conducted to explore the role of TK1 activity in diagnosis and motoring the solid tumors (7, 8). Additionally, TK1 activity lacks the sensitivity and specificity required for accurately diagnosing solid tumors in dogs (6, 9). To overcome the difficulties with TK1 activity-based assays, anti-TK1 antibodies were developed against dog TK1 as an alternative. Early studies demonstrated that TK1 protein-based assays have significantly higher sensitivity in the diagnosis of dogs with solid tumors compared to TK1 activity-based assays (10). Furthermore, canine TK1-ELISA were developed by using the combination of peptide-based monopoly and poly-polyclonal dog TK1 antibodies for diagnosis and therapy monitoring of dog lymphomas (11, 12). Recently, Alertix developed a dual monoclonal antibody-based sandwich TK1-ELISA for diagnosis of dogs with lymphomas as well as solid tumors (13).

Canine C-reactive protein (cCRP) is an acute-phase protein produced by the liver in response to inflammation. In dogs, elevated levels of cCRP have been associated with various conditions, including infection, tissue injury, and cancer (14). The measurement of cCRP levels in the blood may serve as a valuable diagnostic tool for distinguishing between inflammatory and neoplastic conditions (10). The combination of TK1 activity and cCRP levels, as a neoplastic index (NI), has been shown to be valuable for the screening and monitoring of different malignant diseases in dogs (15).

Machine learning and artificial intelligence (AI) have revolutionized human healthcare, where they are widely used for diagnostics, predictive modelling, and personalized treatments (16). In human healthcare, AI techniques such as deep learning and other machine learning algorithms are applied to various types of data, including those from medical imaging, electronic health records (EHR), and wearable sensors. These tools have demonstrated significant success in detecting diseases from images, predicting patient outcomes, and optimizing treatment plans (17). However, the exploration of machine learning in Animal health care is limited. In this study, we used several advanced machine learning techniques, including decision trees, random forests, artificial neural networks, k-nearest neighbors (KNN), gradient boosting modelling (GBM), and regularized regression models (glmnet), which are commonly applied in medical research to build predictive models (18, 19).

The aim of this study was to create an accurate tool to detect different types of dog cancers at an early stage using age, TK1 concentration and cCRP. We aimed to create a machine learning model that utilizes these parameters to classify dogs as positive or negative for cancer and test this model with part of the data not used in the training of the algorithm to validate the model. The secondary aim was to create a user-friendly interface that can be easily applied to the clinical setting to determine the risk of cancer in dogs using the machine learning algorithm.

## Materials and methods

### Serum samples and specimen handling

The study includes 220 samples from dogs with different tumors and 67 healthy dogs. Fifty-three serum samples were from dogs diagnosed with lymphoma (24 T-cell lymphoma and 29 B-cell lymphoma), 167 dogs with different solid tumors [26 from dogs with hemangiosarcoma (HAS), 47 from dogs with histiocytic sarcoma (HS), 26 from dogs with osteosarcoma (OSA), 40 from dogs with mastocytoma (MCT), and 28 from dogs with mammary tumors (MMT)].

These samples were obtained from two sources, i.e., 155 samples were purchased from the Flint Animal Cancer Center (Colorado State University) and 65 samples of different malignancies and the 67 healthy dogs samples were collected at the University Animal Hospital, at the Swedish University of Agricultural Sciences (SLU), Uppsala, Sweden, and stored at −20°C until analysis. The dogs with tumors were naive and have not received any prior treatment for cancer.

As many different tumor types were included, the staging, diagnostics and grading varied according to the tumor entity. In general lymphomas were staged as described in Vail et al. (11) and solid tumors as described in Nguyen et al. (12). Diagnosis were made by histopathology in solid tumors and they were graded as low, moderate or high grade according to current grading schemes applied by the pathology lab used. Lymphomas were diagnosed mainly by cytology examined by board certified clinical pathologists and further diagnosed with PARR (PCR for antigen receptor rearrangements). Grade was predicted as Martini et al. (13). Usually no histopathological confirmation with final grading was made, but accuracy in high/low grade for large blastic vs. small cell indolent lymphomas are fairly good. Cytology along with additional PARR analysis is shown to give high accuracy and align with general clinical practice (14). The group of control dogs were considered healthy based on their medical history, physical examination, hematology, and a basic biochemistry analysis. Most of these subjects were recruited from voluntary blood donor dogs at the University Animal Hospital, SLU, Uppsala, Sweden. Serum samples from dogs with different malignancies and from healthy dogs were collected over a 4-year period (2018–2022). At least 1 mL of blood was drawn from each dog and centrifuged within 1 h of collection. The serum samples were stored at −20°C until analysis.

### Canine TK1 ELISA

The canine TK1 ELISA is a sandwich-based assay using monoclonal anti-TK1 antibodies raised against two different regions of dog TK1as previously described (15). The TK1 protein levels in serum samples were determined by using recombinant dog TK1 as a standard and the concentrations are expressed as pg/mL. The canine TK1 ELISA has a limit of detection (LOD) of 10 pg/mL and the limit of quantification (LOQ) was 20 pg/mL. The cut-off value was set as 2×SD above the mean of TK1 protein levels in healthy dogs. Intra assay CVs with all non-zero calibration points were ≤10% and between-run imprecision was ≤15% at concentrations down to 50 pg/mL.

### TECO canine CRP ELISA

Serum cCRP levels were determined using the TECO^®^ Canine CRP ELISA developed by TECO medical Group in Sissach, Switzerland. This assay is a canine-specific sandwich enzyme-linked immunosorbent assay (ELISA) designed to quantitatively measure cCRP in canine serum. Intra- and inter-assay precision were 4.3 and 6.0%, respectively (16).

### Alertix-Cancer Risk Index

In order to establish the prediction models for individual prognosis or diagnosis, we followed the guidelines for the transparent reporting of a multivariable prediction model. Based on these guidelines, the total patient population was divided into selected 2/3 of the data as the training set and the remaining 1/3 of the data as the test (validation) set. Then we used 10-fold cross-validation to establish machine learning models [decision trees, random forest, GBM (gradient boosting model), and logistic regression] and a deep learning model (ANN) that had the best performance in the training data set. Finally, the test data was used to evaluate the performance of the prediction models by comparing the sensitivity, specificity, positive predictive values, negative predictive values, and area under curve (AUC). Out of these models, the logistic regression model with 10-fold cross validation showed the best performance and was continued for further analysis with validation data set as Alertix-CRI. Only the outcome was reported as results of the best machine learning algorithm. All the machine learning computations were performed by R software 4.2.1 using the caret package (20–22).

### Statistical analysis

Continue variables (TK concentration, age, and cCRP) were summarized by the mean/standard deviation (SD) or median/interquartile range while the categorical variable (cancer type) was summarized by the frequency/percentage. The distributions of cCRP and TK1 protein levels in the healthy and tumor groups were evaluated for normality using the D’Agostino and Pearson omnibus normality test. ROC curves are constructed by using MedCalc software 17.6. A *p*-value less than 0.05 will be considered statistically significant.

## Results

### Study population

The characteristics of the study population were summarized using frequencies or proportions and the medians for categorical and continuous variables, respectively (Table 1). In the scope of this study, a total of 287 samples were collected and the subgroups are summarized in materials and methods. The median age of the healthy group was 48 months, ranging from 12 to 108 months, whereas the median age of the dogs diagnosed with malignant conditions was 108 months, ranging from 18 to 168 months. In the overall population, there were 66 female dogs, 60 male dogs, 66 neutered female dogs, and 92 neutered male dogs (Table 1). Furthermore, within the healthy group, there were 35 male dogs, 22 female dogs, and 10 neutered male dogs, while the diseased group consisted of 44 female dogs, 25 male dogs, 66 neutered female dogs, and 82 neutered male dogs.

**Table 1:** The distributions of TK1 protein concentration, age and CRP in the healthy and malignant groups.

Characteristic	Healthy	B-cell lymphoma	T-cell lymphoma	HAS	HSA	MMT	MCT	OSA
*N* (total No. = 287)	67	29	24	26	47	28	40	26
TK1 Conc (pg/mL)								
Mean (SD)	144 (34)	2,874 (3,267)	324 (423)	390 (383)	532 (679)	188 (89)	207 (171)	274 (364)
Median (IQR)	138 (53)	1,776 (3,084)	180 (207)	224 (276)	274 (326)	164 (106)	138 (112)	173 (160)
Age (months)								
Mean (SD)	45 (21)	107 (38)	94 (33)	111 (34)	100 (26)	120 (19)	110 (27)	96 (38)
Median (IQR)	48 (33)	113 (56)	95 (40)	106 (41)	99 (34)	120 (22)	114 (36)	95 (38)
cCRP (mg/L)								
Mean (SD)	1.6 (1.8)	17.4 (22.7)	10.1 (16)	19 (19.7)	23.4 (21.8)	6.1 (8.4)	3.9 (8.1)	13.9 (15.8)
Median (IQR)	1.0 (1.7)	10.1 (20)	3.2 (10.9)	10.1 (28.2)	11.8 (48.1)	2.7 (7.8)	0.3 (2.0)	7.6 (18.6)

SD, standard deviation; IQR, inter quartile range; B-cell, B-cell lymphoma; T-cell, T-cell lymphoma; HAS, hemangiosarcoma; HSA, histiocytic sarcoma; MMT, malignant mammary tumors; MCT, mast cell tumors; OSA, osteosarcoma.

Table 1 presents the TK1 concentration, age, and cCRP levels across various cancer types and a healthy group. The TK1 concentration in whole cancer group (mean: 684 pg/mL) was significantly higher compared to healthy dogs (mean: 144 pg/mL). The highest concentration was found in B-cell lymphoma (mean: 2,874 pg/mL) whereas the MMT showed a much lower mean TK1 concentration (mean: 188 pg/mL) compared to other tumor types. Age differs across groups, with solid tumor patients generally older (e.g., mammary tumor: mean 119.6 months) than the healthy group (mean: 45.3 months). Median ages also reflect this pattern, with as expected most cancer patients being older than the control group.

However, the higher cCRP levels were found in B-cell lymphomas (mean: 17.4 mg/L) and particularly those with solid tumors like histiocytic sarcoma (mean: 23.4 mg/L) and hemangiosarcoma (mean: 19.0 mg/L) compared to healthy individuals (mean: 1.6 mg/L).

Overall, the data indicate that cancer patients, especially those with B-cell lymphoma and certain solid tumors, have elevated TK1 and CRP levels compared to healthy controls, highlighting differences in inflammatory and tumor marker levels across these groups.

### Canine TK1 ELISA

**Figure 1:**
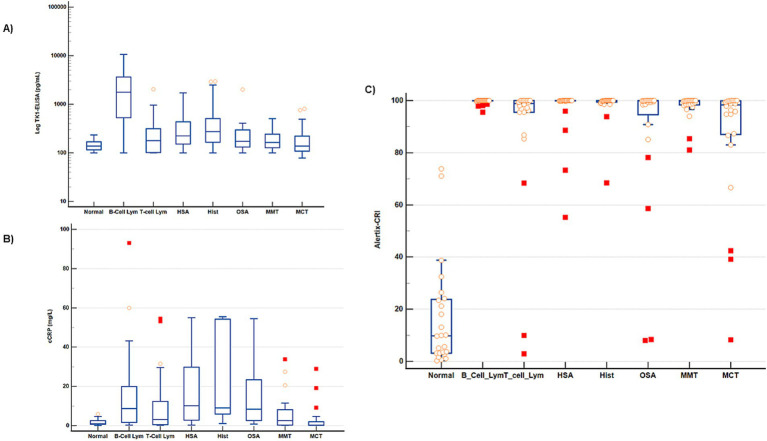
Distribution of TK1 protein levels, cCRP and Alertix-CRI in different groups. Box–Whiskers plots represent the ranges of different groups based on **(A)** log TK1 protein levels **(B)** cCRP **(C)** Alertix-CRI. Tumor subgroups were statistically different (*p* < 0.05) than the control group. The box represents the interquartile range (IQR), with the lower and upper edges corresponding to the first quartile (Q1) and third quartile (Q3), respectively. The line within the box indicates the median value. Solid symbols indicate more extreme outliers, while light symbols indicate less extreme outliers in the box and whisker plots.

Amongst the entire cohort of tumor samples, the receiver operating (ROC) curve analysis revealed an area under the curve (AUC) value of 0.72, with a sensitivity of 54% and a specificity of 95% (Figure 1A). Notably, the positive predictive value (PPV) was determined to be 97%, while the negative predictive value (NPV) reached 40% (Table 2).

**Table 2:** Performance metrics (%) of TK1-ELISA in different tumor subgroups.

Parameter	HAS	HSA	OSA	MCT	MMT	B-cell	T-cell
Cut-off (pg/mL)	200	200	200	200	200	200	200
AUC	0.79	0.83	0.72	0.62	0.70	0.94	0.70
Sensitivity	58.0	70.0	53.0	32.0	33.0	93.0	45.0
Specificity	95.5	95.5	95.5	95.5	95.5	95.5	95.5
PPV	83.3	90.3	87.5	83.3	75.0	90.0	78.6
NPV	85.3	84.2	77.1	67.7	77.1	97.0	83.1

PPV, positive predictive value; NPV, negative predictive value; HAS, hemangiosarcoma; HSA, histiocytic sarcoma; OSA, osteosarcoma; MCT, mast cell tumors; MMT, malignant mammary tumors; B-cell, B-cell lymphoma; T-Cell, T-cell lymphoma.

For the lymphoma group the ROC curve analysis showed an AUC of 0.79 with a sensitivity of 72% at a specificity of 95%. Additionally, the solid tumors group showed an AUC of 0.70 with a sensitivity of 47% at the same specificity. The ROC curve analysis was also conducted on the most common solid tumors subgroups. The specific parameters for each type of studied tumors are shown in Table 2.

### Canine CRP ELISA

When the cCRP was applied to the entire cohort of tumor samples, the ROC curve analysis demonstrated a similar area under the curve (AUC) of 0.70 compared to the canine TK1 ELISA with a sensitivity of 50% at a specificity of 95% (Figure 1B). In contrast to the canine TK1 ELISA, both the lymphoma and solid tumors groups exhibited slightly lower AUC values of 0.71. Moreover, the sensitivity for these groups was 51% at a specificity of 95%, (data not shown).

### Alertix-Cancer Risk Index

**Figure 2:**
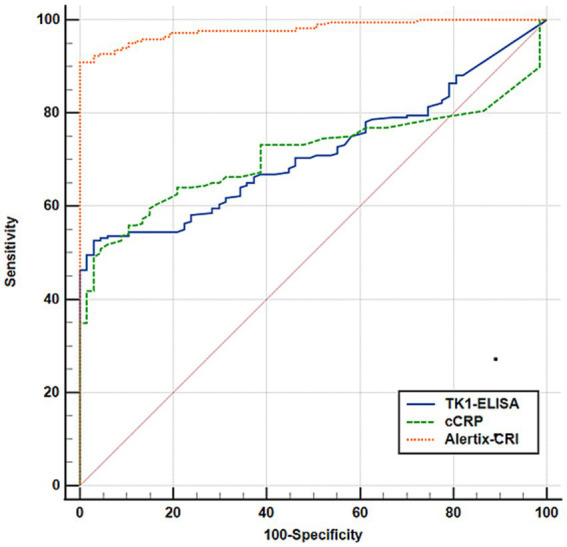
Overall comparison of receiver operating characteristic (ROC) curves between assays. ROC curve comparison of TK1-ELISA, cCRP, and Alertix-CRI for differentiation of the healthy controls from the tumor group. Blue line represents the canine TK1 ELISA model, green line represents the cCRP model and orange line represents Alertix-CRI.

The Alertix-CRI is a model based on tenfold cross-validated logistic regression, which includes the combination of TK1 concentration, age and cCRP levels. Since the results of the cross validated logistic regression is more explainable (by providing the odds ratios) and because of the small sample sizes, we only report the results of the cross-validated in Table 3. The optimal parameters for the GBM are also reported in Supplementary Table 1. The distribution of Alerix-CRI in different tumor subgroups are shown in Figure 1C. Table 3 compares the performance metrics for different cancer types, including all cancers, B and T-cell lymphoma, and several solid tumors. Sensitivity is consistently high across all cancer types, with most reaching 100%, indicating high capacity of the model to correctly identify the positive cases. Furthermore, a comparison of ROC curves between TK1-ELISA, cCRP and Alertix-CRI clearly demonstrated the higher performance of the Alertix-CRI (Figure 2). Different specificities were observed and cancers like T-cell lymphoma and osteosarcoma showed lower values (88 and 67%, respectively) compared to other tumors. The positive predictive value (PPV) and negative predictive value (NPV) are above 90%, suggesting very accurate predictions. Precision and recall are similarly high, with *F*_1_-scores close to 100% for most cancers, reflecting the balance between precision and recall. The AUC values are also high, indicating excellent overall model performance, although a few cases like osteosarcoma (80%) show slightly lower discriminatory ability.

**Table 3:** Performance metrics (%) of Alertix-CRI in different tumor subgroups.

Metric	All cancers	B-cell cell lymphoma	T-cell cell lymphoma	HAS	HSA	MMT	MCT	OSA
AUC	98	99	100	96	92	100	94	80
Sensitivity	90	100	100	100	95	100	95	100
Specificity	97	88	100	83	95	100	80	67
PPV	90	95	100	95	95	100	90	91
NPV	97	100	100	100	92	100	89	100
Precision^a^	90	95	100	95	95	100	90	91
Recall^b^	90	100	100	100	95	100	95	100
*F*_1_-score^c^	90	98	100	98	95	100	93	95

PPV, positive predictive value; NPV, negative predictive value.

a Precision: The ratio of true positive results to the total number of positive results predicted by the model.

b Recall: The ratio of true positive results to the total number of actual positive cases.

c *F1-score: The harmonic mean of precision and recall.*

## Discussion

In the contemporary era of personalized medicine, health screening determinations no longer exclusively rely on single indicators. Advanced prediction software based on optimized algorithmic models has emerged as a superior method for guiding screening outcomes (17). The findings of this current study indicate that the integration of serum thymidine kinase 1 (TK1), C-reactive protein (CRP), and age of dogs as represented here by the machine learning model based Alertix-CRI exhibits a stronger predictive capacity for the detection of various malignancies compared to individual biomarkers (16, 18). This investigation leverages machine learning techniques to assess the three-dimensional combination of clinical parameters for predicting the presence of tumors in veterinary medicine. While previous studies have evaluated multiplex markers for canine mammary tumor detection, our study uniquely integrates machine learning to enhance predictive accuracy and provide novel insights into tumor detection (19, 23). The criteria to select the best diagnostic model based on the sensitivity, specificity, and the predictive values. Overall, the Alertix-CRI was able to identify 93% of all malignant samples in this population of dogs.

The primary outcome of this study is the noteworthy observation that Alertix-CRI exhibited exceptional precision to identify different types of malignancies including the most common and aggressive tumors, e.g., mastocytoma, mammary tumors, lymphoma and hemangiosarcoma, as evidenced by an area under the curve (AUC) of 0.97, surpassing the previously reported value of 0.88 (18). In contrast to other non-invasive tumor biomarkers, such as The Nu. Q^®^ Vet Cancer Test, which exhibited an AUC of 69% and a sensitivity of approximately 50% for all the cancers they studied in their cohorts (20), our model demonstrated superior performance. The AUC for Alertix-CRI was 98%, with a remarkable sensitivity of 90%. Moreover, we achieved these outcomes while maintaining high specificity across the various types of cancers. Ensuring high specificity is crucial in the screening program due to the potential financial and psychological burdens associated with false positives, as well as the potential harm that can result from unnecessary diagnostic interventions.

Cancer is a complex disease characterized by a multifactorial etiology, encompassing contributions from both genetic and environmental factors. Several studies have investigated the relationship between animal cancers and environmental influences, with some demonstrating correlations between specific cancers and environmental factors, the others have shown itis associations with specific animal breeds (21, 22). The risk of developing cancer increases with age. This association is thought to be due to cumulative exposure to environmental factors, accumulated genetic mutations over time, and the gradual decline of cellular repair mechanisms (24). The aging process itself can also contribute to the development of cancer by influencing cellular senescence, DNA damage, and immune system function (24). A recent study involving a cohort of over 3,000 dogs aimed to ascertain the appropriate age to commence cancer screening tests. The findings indicated that the optimal age to initiating cancer screening was 7 years for all dogs and 4 years for a breed with lower median age (25). Thus, in the current feasibility phase of our Alertix-CRI designed for cancer detection in dogs, we have integrated the age of the animals alongside the specific tumor biomarker TK1 and the inflammatory biomarker cCRP.

The sensitivity of the Alertix-CRI across various cancer types ranged from 90 to 100%, demonstrating its robustness in correctly identifying malignant cases. Particularly, T-cell lymphoma and solid tumors like mammary tumors and osteosarcomas showed perfect sensitivity (100%). The Alertix-CRI also performed well in terms of specificity, ranging from 83 to 100%, although certain tumors, such as hemangiosarcoma and osteosarcoma, had lower specificity values (83 and 67%, respectively).

For precision (positive predictive value), the system maintained high values across the board, with all cancers showing precision between 90 and 100%, indicating that when the Alertix-CRI predicted cancer, it was highly accurate. Similarly, the negative predictive value remained high, reaching 100% for most cancer types, ensuring that healthy cases were rarely misclassified as cancerous. The *F*_1_-score, which balances precision and recall, was consistently high (ranging from 90 to 100%), reaffirming the diagnostic tool’s reliability in distinguishing between healthy and malignant cases.

The area under the curve (AUC) values further underscore the Alertix-CRI excellent performance, with most cancers achieving AUCs above 95%, indicating near-perfect classification ability. Solid tumors such as hemangiosarcoma (96%) and mastocytomas (94%) showed slight dips in AUC, though the Alertix-CRI still performed effectively. Even though the TK1 concentration and cCRP alone have an AUC of 0.75 but when we combine TK1 with cCRP the AUC significantly increased to 0.89 which clearly indicate the complimenting of these two biomarkers in cancer detection. As described earlier CRPs nonspecific nature makes it an unreliable tumor biomarker on its own, as it cannot distinguish between inflammation caused by cancer and other inflammatory conditions. To accurately evaluate the role of CRP in early cancer detection, it is essential to consider the complex relationship between inflammation and cancer. Chronic inflammation promotes the development of dysplasia and ultimately predisposes to cancer. Additionally, inflammation plays an essential role at each stage of cancer development, correlating directly with the degree of associated inflammation. Combining CRP with other biomarkers, such as thymidine kinase 1 (TK1), can enhance the sensitivity for early tumor detection. In summary, while CRP is a valuable marker for inflammation, its nonspecific nature necessitates the inclusion of additional biomarkers, such as TK1, to improve the accuracy of early cancer detection. Further inclusion of TK1 in the index reduces the contribution of CRP increase during inflammatory conditions. This approach helps distinguish cancer-related positivity from inflammation-induced elevation without the need for a separate control group of dogs with inflammatory conditions but no tumors. Earlier published studies showed that this approach of CRP and TK1 increase the specificity of cancer detection (16, 26). However, in further validation studies with CRI it is reasonable to include dogs with inflammatory conditions as well. Finally, age is one of the predisposing factors for cancer and addition of age factor to TK1 and cCRP increased the AUC further from 0.89 to 0.98 in differentiation of tumor dogs from healthy (Supplementary Figure 1).

This study had two key methodological strengths. First, we applied several advanced machine learning algorithms to compare their accuracies using cross-validated data. By rigorously testing these models, we ensured a robust comparison of their predictive performances.

Among the various models tested, logistic regression based Alertix-CRI demonstrated the most consistent and reliable accuracy in cross-validation, leading to its selection for further analysis.

Second, after selecting logistic regression as the optimal model, we further evaluated its performance on an independent test dataset. This external validation step is critical, as it ensures that the model generalizes well to new, unseen data, enhancing its practical applicability. We also included the cross-validated logistic regression as a conventional statistical method for comparison. Unlike logistic regression, machine learning models do not rely on statistical assumptions (such as linearity or uncorrelated predictors) and can handle complex interactions among predictive factors without the need for pre-specifying these interactions. The outcomes from this research could with adaption also be used for other species and therefore improve animal welfare overall. However, the age group of healthy dogs is one of the limitations for this study due to the complications associated with serum samples from aged healthy dogs. Most studies have a skewed median age between normal controls and tumor groups, but there are exceptions (23). To our knowledge there is no such study at this scale in canine oncology, and thus it may enhance the possibilities for future research and AI-applications within animal welfare. Further studies with prospective validation of the Alertix-CRI with more inclusion of aged healthy dog population may enhance the clinical applications of biomarkers in veterinary medicine.

## Data Availability

The raw data supporting the conclusions of this article will be made available by the authors, without undue reservation.
