# Supplementary figures and images for: A novel cross-validated machine learning based Alertix-Cancer Risk Index for early detection of canine malignancies
Source: Front Vet Sci. 2025 Apr 25;12:1570106. doi: 10.3389/fvets.2025.1570106 (PMC12061885; doi:10.3389/fvets.2025.1570106)

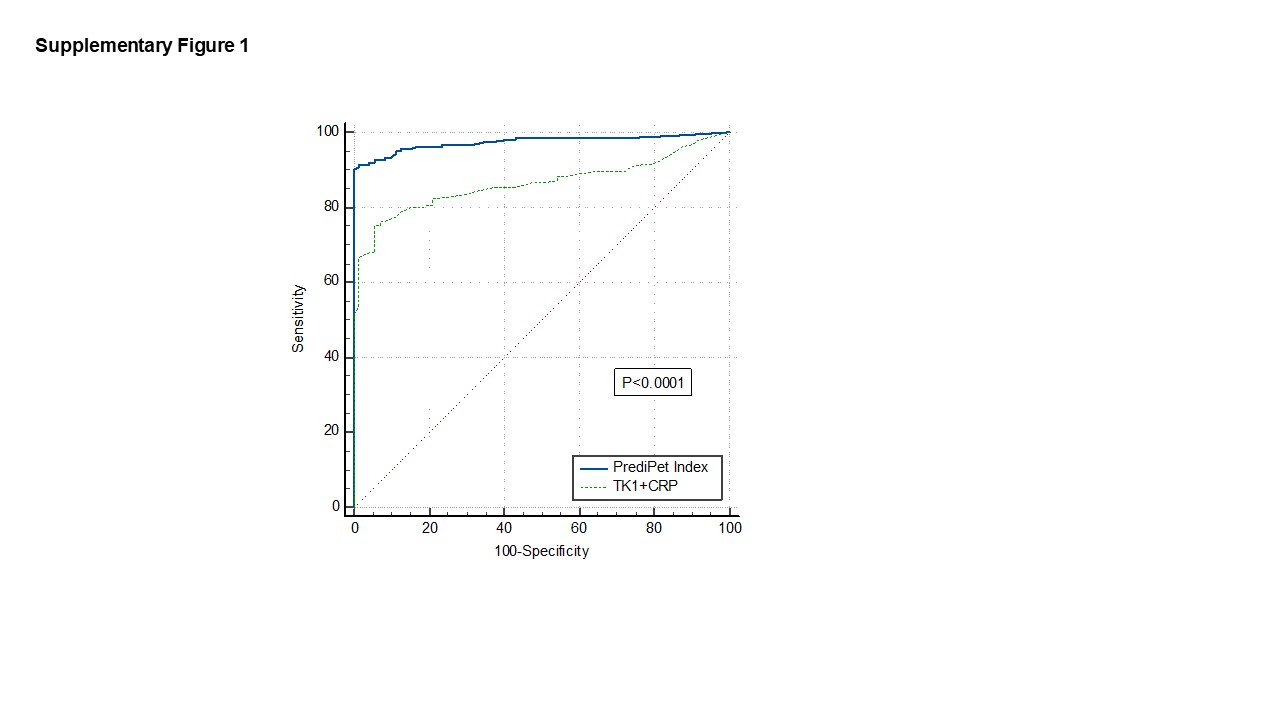

Supplement: Supplementary file 1 [file Image_1.jpg]
