# Supplementary material for: A novel cross-validated machine learning based Alertix-Cancer Risk Index for early detection of canine malignancies
Source: Front Vet Sci. 2025 Apr 25;12:1570106. doi: 10.3389/fvets.2025.1570106 (PMC12061885; doi:10.3389/fvets.2025.1570106)
Supplement: Supplementary file 2 [file Table_1.docx]

# GBM Model Summary Table

| Cancer Type | Number of Trees | Interaction Depth | Shrinkage | Min Obs in Node |
| --- | --- | --- | --- | --- |
| All Cancer | 200 | 3 | 0.2 | 10 |
| Blood cancer (B cell lymphoma) | 50 | 2 | 0.20 | 10 |
| Blood cancer(T cell lymphoma) | 50 | 3 | 0.05 | 10 |
| Solid tumors (Hemangiosarcoma) | 200 | 2 | 0.01 | 10 |
| Solid tumors (Histiocytic sarcoma) | 50 | 1 | 0.20 | 10 |
| Solid tumors (Mammary tumor) | 50 | 1 | 0.05 | 10 |
| Solid tumors (Mstocytoma) | 50 | 7 | 0.10 | 10 |
| Solid tumors (Osteosarcoma) | 50 | 5 | 0.30 | 10 |
